# Bridging the ‘Two Cultures’ of Research and Service: Can Complexity Theory Help? Comment on "Experience of Health Leadership in Partnering With University-Based Researchers in Canada – A Call to ‘Re-imagine’ Research"

**DOI:** 10.15171/ijhpm.2019.89

**Published:** 2019-10-28

**Authors:** Trisha Greenhalgh

**Affiliations:** University of Oxford, Oxford, UK.

**Keywords:** Health System, Knowledge Translation, Complexity, Research-Service Gap

## Abstract

This commentary addresses Bowen et al’s empirical study of perspectives of Canadian healthcare staff towards research and their call for multi-faceted action to improve misalignments in the system. This commentary argues that tensions and misalignments between research and service are inherent and can never be eradicated. Building on previous work by Lanham et al, I propose seven principles of complexity which may help to develop system capacities that will help bridge the research-service gap: acknowledge unpredictability, recognise selforganisation, facilitate interdependencies, encourage sensemaking, attend to human relationships, develop adaptive capabilities in staff, and harness conflict productively.

Bowen et al’s important and well-written paper, which reports interviews with senior health personnel across Canada, reveals some new but unsurprising findings about why healthcare organisations and their members have difficulty engaging with research.^1^ The system is complex; stakeholders are multiple and their goals and values differ; structures, interfaces and incentives are misaligned (and, at the level of individual healthcare staff, often perverse); and – despite a laudable call for “multi-system action” – there are no easy answers.

The word “complex” or “complexity” recurs many times in the paper, sometimes in the phrase “complex adaptive systems.” Could a complexity lens help to theorise the paper’s findings and sharpen the recommendations? I think it can.^2^

Cohn et al define a complex adaptive system as “*a dynamic and constantly emerging set of processes and objects that not only interact with each other, but come to be defined by those interactions.* ”^3^ Characteristics of such systems include fuzzy (that is, indistinct and porous) boundaries; actors who follow “simple rules” as they respond and adapt to local information and circumstances; and enmeshment with other systems with which the system interacts adaptively and co-evolves.^4-6^ The extent to which elements of the system (both individual actors and organisations) are able to adapt is also the extent to which the system can learn and change. Complexity is a feature of the system(s), not merely a characteristic of interventions.^7,8^ Change is ever-present; mess and uncertainty cannot be eliminated. You cannot predict a complex system; the best you can do it observe it carefully and adapt to what emerges.

Importantly, all these features are an *inherent* property of complex systems. They cannot be fixed; they must be lived with. Adaptiveness is an under-recognised and under-rated feature of complex systems. Articulations and workarounds by creative, motivated people may help square the circle and deliver the system’s multiple goals.

In a paper on spread and sustainability in healthcare, Lanham et al recommend the following rules of thumb for making progress in conditions of complexity^9^:

- *Acknowledge unpredictability* : contemplate multiple plausible futures; tailor activities to local context and view surprises as opportunities;
- *Recognise self- organisation* : expect designs to be modified, perhaps extensively, as they are taken up in different settings; proactively capture data and feed it into the adaptation process;
- *Facilitate interdependencies* : develop methods to assess the nature and strength of interdependencies; surface and study the nature of interrelationships, reinforcing existing ones where appropriate and facilitating new ones;
- *Encourage sensemaking* : engender a culture in which participants ask questions, admit ignorance, explore paradoxes, exchange different viewpoints and reflect collectively.

Our own team added the following to Lanham et al’s suggestions^9^:

- *Attend to human relationships* : since embedding innovation requires people to work together to solve emergent problems using give-and-take and “muddling through;”
- *Develop adaptive capabilities* : train staff (in both the research and service sectors) not merely to complete tasks as directed but to ‘tinker’ with processes and tools make judgements when faced with incomplete or ambiguous data;
- *Harness conflict productively* : view conflicting perspectives as the raw ingredients for multifaceted solutions, since there is rarely a single, right way of addressing a complex problem.

These seven principles align well with the recommendations of Bowen et al (see ‘Key Messages’ in their paper). Their call for a broader definition of research (eg, to encompass applied and evaluative designs), along with a plea to research funders for more agile and responsive funding resonates with the principle of capturing emerging data and feeding it into system responses. Their call for more and better education of researchers explicitly includes “understanding complexity” and the need to develop the interpersonal skills and attitudes needed for effective cross-sector working. Finally, their call for leadership to support intersectoral partnership-building underlines the focus in complex adaptive systems on the relationships between the actors, not just the actors themselves.

In conclusion, an awkward tension between service logic and research logic will – in my view – always exist, and that tension will be felt most acutely in resource-constrained circumstances. At times of stress there is always a retreat from integration. To make the best of this situation, we – that is, researchers, health service workers and our respective sectors – need to build relationships, maintain dialogue and muddle through.

## Ethical issues

Not applicable.

## Competing interests

Author declares that she has no competing interests.

## Author’s contribution

TG is the single author of the paper.
